# Supplementary material for: Are carbon emissions trading and green financial instruments synergistic? -Comprehensive quantitative research based on content analysis
Source: PLoS One. 2024 Mar 7;19(3):e0298601. doi: 10.1371/journal.pone.0298601 (PMC10919601; doi:10.1371/journal.pone.0298601)
Supplement: S1 Data — (ZIP) [file pone.0298601.s001.zip › Data/地级市Z_F工作报告.zip!/─┌├╔╣┼/║⌠┬╫▒┤╢√╩╨/└┤╫╘╣1⁄2╓┌║┼í╛┬φ┐╦╩2╛▌═°í┐_2018.docx]

各位代表：

现在，我代表市人民政府向大会报告工作，请予审议，并请市政协委员和列席会议的同志提出意见。

一、过去五年工作回顾

过去的五年，在自治区党委、政府和市委的正确领导下，市政府团结带领全市各族人民，牢牢把握稳中求进工作总基调，统筹做好稳增长、促改革、调结构、惠民生、防风险各项工作，真抓实干，攻坚克难，经济社会发展取得了新进展、新成就，为决胜全面建成小康社会奠定了坚实基础。

（一）综合经济实力跃上新台阶。过去的五年，是我市经济总量不断壮大、质量效益同步提升的五年。到2017年底，按可比口径计算，预计地区生产总值达到1770亿元，年均增长7.3%；一般公共预算收入达到85.3亿元，年均增长2.5%；城镇常住居民人均可支配收入达到31196元，年均增长8.7%；农村牧区常住居民人均可支配收入达到13606元，年均增长9.9%；社会消费品零售总额达到645亿元，年均增长9.7%。煤炭、电力、木材、有色金属、农畜产品加工五个产业年产值超百亿元。传统服务业加速发展，现代服务业不断成长，旅游影响力和品牌竞争力明显提升，旅游人数和旅游收入年均分别增长11.2%和23.9%。金融助推实体经济成效显著，社会融资总规模年均增长15.4%，直接融资能力实现新突破。

（二）生态文明建设取得新进展。过去的五年，是我市整体生态环境持续向好、生态文明制度建设力度加大、全社会生态环保意识显著增强的五年。全市累计造林510.5万亩，活立木蓄积量达到2.7亿立方米，森林覆盖率达到51.4%。国有林区天然林商业性停伐政策全面落实。草原生态补助奖励累计发放26.3亿元，落实禁牧和草畜平衡1亿亩以上。沙区综合治理累计投入5.8亿元，治理面积329万亩，荒漠化和沙化面积实现“双减少”。呼伦湖综合治理成效明显，水域面积稳定在2050平方公里左右，水质及周边生态环境明显改善，依法治湖实现历史性突破。最严格水资源管理制度得到落实，河长制全面推行。矿山治理投入资金4.6亿元，完成治理面积6.8万亩。高标准基本农田建设完成投资13.7亿元，累计治理面积95.4万亩。海拉尔西山自然保护区、海拉尔河、伊敏河生态修复工程取得阶段性成果。大气、水、土壤污染防治工作取得积极进展。自治区下达的节能节水减排任务全部完成。国家主体功能区建设试点、自然资源资产负债表编制试点、领导干部自然资源资产离任审计试点等工作全面推进。

（三）转方式调结构迈出新步伐。过去的五年，是我市发展方式由粗放型增长向集约型增长转变，产业结构不断优化的五年。绿色发展理念深入人心，经济发展对资源的依赖程度逐步降低。三次产业结构由2012年的17.9：47.1：35优化为15:45:40。产业内部结构优化调整提速。农畜产品加工转化率五年提高20.8个百分点，达到58%。传统工业转型升级步伐加大，战略性新兴产业不断成长。民营经济持续发展，中小企业素质提高，非公经济增加值占地区生产总值比重由51.7%提高到54.3%。呼伦贝尔肉业集团“中荣”商标荣获中国驰名商标，呼伦贝尔农垦集团“苍茫谣”牌芥花油荣膺首届十佳国产食用植物油创新品牌。“三去一降一补”扎实推进,累计退出105万吨煤炭和7万吨钢铁产能，消化商品房库存150万平方米，直供电交易降低企业成本超过2亿元。

（四）城乡面貌发生新变化。过去的五年，是我市城镇规划建设管理水平稳步提高、农村牧区基础设施日趋完善的五年。中心城、重点镇和新区建设统筹推进。市政基础设施累计投资369亿元，完成城市道路、广场、绿地、污水处理等项目1861个。新开工建设各类保障性安居住房22.7万套，惠及59万群众。首个地方性法规《呼伦贝尔市城市市容和环境卫生管理条例》颁布实施。城市管理执法体制改革全面启动。中心城区荣获“自治区园林城市”称号。扎兰屯市和阿荣旗荣获“国家园林城市”称号。莫尔道嘎镇、柴河镇荣获“中国特色小镇”称号。扎兰屯市、大杨树镇被列入国家新型城镇化试点。公共服务向基层全面延伸，行政村全部实现通水、通电、通路。基础设施建设力度加大，公路、铁路、民航五年投入420.6亿元，新增公路里程6586公里，新增铁路里程59.6公里，新增机场4座。滨洲铁路电气化改造竣工，阿荣旗—扎兰屯铁路通车，海拉尔—满洲里城际列车试运行，齐海满客运专线列入国家中长期铁路发展规划。海拉尔东山机场旅客年吞吐量突破200万人次，根河通用机场投入运营，扎兰屯支线机场、新右旗通用机场实现通航，莫旗、阿荣旗通用机场竣工，全市一小时航空圈初步形成。水利建设五年投入40亿元，扬旗山水利枢纽工程竣工，雅鲁河治理主体工程完工，Z866、扎敦水利枢纽等项目积极推进。信息通信网实现全覆盖，宽带网络实现村村通。城乡电网五年投入111.4亿元，电网长期薄弱、发展滞后局面明显改善。

（五）改革开放实现新突破。过去的五年，是我市改革攻坚力度持续加大、开放水平逐步提高、发展动力稳步增强的五年。经济、生态、文化和社会领域改革任务累计完成285项，精简、取消、下放行政审批事项539项。政府机构改革及“三单”梳理工作如期完成，“放管服”改革向纵深迈进，商事制度改革成效明显。呼伦贝尔农垦集团、国有林区和地方国有林场改革取得阶段性成果。农村牧区改革积极推进。医药卫生体制改革进一步深化，公立医院全部取消药品加成。职称制度、市直属国有企业负责人薪酬制度、机关事业单位养老保险制度、公务用车改革有序实施。不动产统一登记制度得到落实，登记发证工作实现全覆盖。呼伦贝尔市被国家旅游局确定为首批旅游业改革创新先行区。呼伦贝尔城投集团多元化经营稳步拓展。中心城区公交一体化改革实现突破。积极融入“一带一路”建设和新一轮东北振兴战略，呼伦贝尔中俄蒙合作先导区上升为国家战略。满洲里国家重点开发开放试验区建设全面推进，综合保税区建设步伐加快。口岸基础设施建设和互联互通项目进展良好，整体通关能力进一步提高。五年进出口总额累计完成146.7亿美元，年均增长9.4%。引进国内（市外）资金3415亿元。

（六）民生福祉得到新改善。过去的五年，是我市人民群众获得感、幸福感明显增强的五年。民生领域公共财政投入持续增加，累计支出1380.4亿元，年均增长10.7%。投入各类扶贫资金41.9亿元，实现精准脱贫17万人，贫困发生率由24.5%下降到2.6%。发放各类惠农惠牧补贴74.8亿元。创业就业工程扎实推进，城镇累计新增就业17万人，农牧民转移就业38.1万人，高校毕业生就业5.3万人，零就业家庭实现动态清零，城镇登记失业率控制在4%以内。基本养老保险、城乡基本医疗保险制度实现政策全覆盖。城镇居民医疗保险财政补助每人达到450元。社会救助体系不断完善，城乡低保标准分别达到每人每月530元和每年3800元，分别提高26.8%和36.1%。

（七）社会事业再上新水平。过去的五年，是我市基本公共服务水平明显提高的五年。科技领域成果丰硕，先后组建10个院士工作站，创建2个国家级众创空间，成立呼伦贝尔生态产业技术研究院。各级各类教育协调发展，办学条件逐步改善，教育质量持续提升，13个旗市区通过国家义务教育发展基本均衡县验收，组建了扎兰屯职业学院。公共文化服务体系更加健全，文化产业良性发展。公共卫生服务能力显著增强，基层卫生服务体系日益完善，计划生育政策得到全面落实。群众体育深入普及，竞技体育水平不断提高。民族宗教政策得到贯彻落实。“光明行”社会公益活动积极开展，实施免费复明手术2479例。审计、统计、广播电视、新闻出版、国资监管、人防、外事侨务、供销、气象、地震、档案史志、关工委、老龄委等工作都取得新进展。

（八）政府自身建设有了新提升。过去的五年，是我市各级政府执行力、公信力、服务能力和社会治理能力全面提高的五年。依法治市进程加快，社会治理体系逐步完善。累计办理人大代表议案5件、建议628件、政协委员提案1084件。行政执法和公正司法水平明显上升，我市荣获国家“六五”普法中期先进城市称号。政务公开工作力度加大，行政监察、审计监督和社会监督作用得到充分发挥。党的群众路线教育实践活动、“三严三实”专题教育和“两学一做”学习教育取得实效，政府系统工作作风明显转变。全国双拥模范城创建实现四连冠。国防动员和后备力量建设不断加强。基层政权和社区建设稳步推进。平安呼伦贝尔创建活动扎实开展。食品药品监管持续加强。安全生产事故起数和死亡人数实现双降。妥善解决群众合理诉求，累计化解信访积案4213件。我市荣获国家“社会管理综合治理优秀市”称号。

各位代表，2017年，我市全力以赴稳增长，全面加强生态建设，努力稳住工业经济，加快农牧林产业化发展，提升现代服务业水平，推动城乡协调发展，深化改革开放，提高社会建设水平，保持了经济社会持续健康发展。同时，主动适应经济发展新常态，降低资源开发强度，调减指标任务，挤出数字水分，克服了经济下行、有效投资下滑、持续干旱等困难，守住了发展底线、民生底线、生态底线，整体经济运行质量和效益不断提高，实现了稳中有进、稳中向好的预期目标。按可比口径计算，预计地区生产总值增长3.5%左右，一般公共预算收入增长2.6%，规模以上工业增加值增速4%左右，限额以上固定资产投资与上年基本持平，社会消费品零售总额增长7.5%左右，城乡居民人均可支配收入分别增长8%和8.5%。先行性指标持续增长，全社会用电量增长5.2%，其中工业用电量增长5.7%；铁路货物发送量增长12.9%，公路货物周转量增长14%；贷款余额增长10.2%，超过存款余额增速5.2个百分点。全市亿元以上重点项目当年完成投资347.7亿元。阜丰技改、晟通公司100万吨甜菜制糖、伊赫塔拉肉羊精细加工等项目进展较快。鄂伦春嘎仙洞、根河敖鲁古雅等具有民族和历史文化特色的景区陆续开园。“冷极号”旅游列车启程运营。呼伦贝尔市荣获“全国十佳冰雪旅游城市”称号，获得创建全国文明城市提名资格。中心城区草原音乐名城、冰雪运动名城、生态旅游名城的“三城”建设全面启动。清理环境卫生、清理违章建筑、清理交通秩序的“三清”治理初见成效。国务院正式批复额布都格、阿日哈沙特公路口岸为双边性常年开放公路运输口岸。一年来，我们高质量承办了全区旅游发展大会、全区第九届少数民族传统体育运动会、第十三届海拉尔·中俄蒙经贸洽谈暨产品展销会和内蒙古冬季旅游那达慕，开展了自治区成立70周年系列庆祝活动。我们以高度的政治自觉，全面加强了中央、自治区环保督察整改和巡视整改，呼伦湖综合治理、北方药业等问题整改取得阶段性成果。

各位代表！过去五年取得的成绩，是自治区党委、政府和市委坚强领导的结果，是市人大、市政协有效监督、鼎力支持的结果，是全市各族干部群众拼搏进取、团结奋斗的结果，是各民主党派、人民团体、驻呼军警部队指战员及社会各界广泛参与的结果。在此，我代表市政府，向关心和支持呼伦贝尔市发展的同志们、朋友们，表示衷心的感谢!

在肯定成绩的同时，更要清醒地看到我市发展中存在的问题：一是落实新发展理念的思路不够宽、办法不够多、效果不够好。二是经济结构不优，新兴产业发育缓慢，产业转型升级任务艰巨。三是基础设施短板较多，城乡收入差距较大，发展不平衡不充分问题比较突出，基本公共服务均等化还需下大力气。四是破坏生态、污染环境等现象时有发生，生态文明建设特别是制度建设任重道远。五是社会治理面临许多新课题，社会建设存在薄弱环节。六是少数干部担当意识不强，执行能力不足，工作状态不佳，作风建设还需要进一步加强。对此，我们一定要正视问题，不遮掩、不回避、不懈怠，采取坚决有力措施加以解决。

二、今后五年主要工作

今后五年，是我市推动全面转型升级的关键期，也是全面深化改革的攻坚期，到2020年要全面建成小康社会，新征程面临新挑战，新时代孕育新机遇，我们必须保持战略定力，紧紧围绕市四次党代会确定的“两区三地一家园”目标定位，坚定发展信心，团结奋斗，开拓进取，全力推动经济社会持续健康发展。

今后五年政府工作的总体要求：全面贯彻党的十九大精神，以习近平新时代中国特色社会主义思想为指导，深入落实自治区第十次党代会和市第四次党代会精神，坚持稳中求进工作总基调，践行新发展理念，按照高质量发展的要求，统筹推进“五位一体”总体布局，协调推进“四个全面”战略布局，以供给侧结构性改革为主线，统筹推进稳增长、促改革、调结构、惠民生、防风险各项工作，全力推动质量变革、效率变革、动力变革，坚决打好防范化解重大风险、精准脱贫、污染防治三大攻坚战，坚决守好发展、生态和民生三条底线，加快美丽发展、转型升级，扩大开放，强化创新，决胜全面建成小康社会，努力开创呼伦贝尔现代化建设的新局面。

今后五年经济社会发展的预期目标：按可比口径计算，地区生产总值年均增长5%左右;一般公共预算收入年均增长5%以上;限额以上固定资产投资年均增长7%左右;社会消费品零售总额保持自治区平均水平;城乡居民人均可支配收入年均分别增长7.5%左右。

从2018年开始，我市全面推行绿色发展指标体系，主要预期目标：草原综合植被覆盖度稳定在70%以上；新增活立木蓄积量1.2亿立方米以上；主要河流湖泊功能区水质达标率达到72%；污水集中处理率达到97%；可治理沙地土地治理率、受污染耕地安全利用率达到自治区目标；城市空气质量优良天数比例不低于97%。

绿色发展指标体系涵盖了资源利用、环境治理、生态保护、增长质量、绿色生活等方面。推行绿色发展指标体系，对不同类型地区进行差异化考核，是落实国家主体功能区战略、加快生态文明制度建设、筑牢祖国北疆重要生态安全屏障的必然要求，是走美丽发展之路的重要支撑和保障。

为实现上述目标，新一届政府要努力做到：

——生态环境更加美丽。坚定不移推动生态文明建设，牢固树立“绿水青山就是金山银山”的理念，像对待生命一样对待生态环境，统筹山水林田湖草系统治理。以打造全国可持续发展的生态环境保护建设示范区为目标，全面推进主体功能区建设，形成节约资源和保护环境的空间格局、产业结构、生产方式、生活方式。实施生态修复治理和生态系统保护工程。编制草原、森林、河湖、黑土地中长期保护规划和呼伦贝尔生态系统整体保护规划。实施好呼伦贝尔大草原、大兴安岭森林、河湖水系、黑土地保护治理工程，通过政府购买社会服务等方式推进沙区综合治理，以呼伦湖治理为重点，推动生态建设由点上治理向全域治理转变，由工程治理向科学管护转变，切实保护好呼伦贝尔完整的生态系统。完成生态保护红线、永久基本农田、城镇开发边界三条控制线划定任务。打好污染防治攻坚战。深入推进大气、水、土壤污染防治行动并取得重大成效。强化生态环境监管。推动建立市场化、多元化生态补偿机制。统一行使监管城乡各类污染排放和行政执法职责。生态文明制度体系逐步健全，崇尚生态文明、共促绿色发展的理念深入人心。通过5年的努力，让呼伦贝尔这片“绿色净土”更加山清水秀、林茂草盛、天蓝云白，人与自然和谐共生。

——经济发展更加高效。坚持质量第一、效益优先，全面推动高质量发展。以供给侧结构性改革为主线，以打造原生态、国际化、全域游高端旅游目的地、绿色有机农畜林产品生产加工输出基地和清洁能源生产加工输出基地为目标，推动产业转型，着力构建存量绿色化、增量高端化的产业发展新格局，一二三产业融合发展取得新成效。做精一产，加快农牧业现代化进程。以优化布局、调优结构为重点，以名优特精为方向，以提高供给质量和综合效益为核心，以培育新型农牧业经营主体、构建企业与农牧民科学合理的利益联结机制为纽带，实施龙头带动、品牌培育、科技支撑行动，启动草业提升、乳业振兴、薯业崛起计划，努力将我市打造成国家级生态草牧业示范区、国家级原生态草原及游牧文化保护区、国家级特色农畜产品优势区、内蒙古一二三产业融合先行区、东北亚农产品国际商贸物流中心。做优二产，加快工业转型升级。推动煤电化等传统资源能源型产业进行技术改造、延长产业链条。培育壮大蒙中医药、节能环保、清洁能源、生物科技、大数据、云计算等战略性新兴产业，绿色产业成为新的增长点。做强三产，加快现代服务业发展，大力提高服务业比重和竞争力。推进国家级旅游业改革创新先行区建设，实现文化、体育、民族、民俗与旅游深度融合，将旅游业培育成新的支柱产业。发展壮大商贸物流、金融会展、健康养老等现代服务业。通过5年的努力，形成质量更高、效益更好的多元发展、多极支撑的现代经济体系。

——城乡区域更加协调。加快以人为核心的城镇化进程，补齐基础设施短板，推动全市城镇体系建设上水平。坚持中心城区优先发展，完善市区共建机制，加大市区共建力度，增强中心城区辐射带动能力。以发展旗市所在地为重点，优化生产力布局，推进产城融合，壮大县域经济，着力打造一批规模适度、宜居宜业宜游的城关镇、特色乡镇，加快构建有呼伦贝尔特色的生态型城镇体系。加快推进市域内公路、铁路、航空、水利、电力、信息通信等基础设施建设，打造一小时航空圈，形成方便快捷、立体化的交通运输体系，发展保障能力显著增强。大力实施乡村振兴战略，坚持农业农村优先发展，建立健全城乡融合发展体制机制和政策体系，统筹推进农村牧区各项建设，走城乡融合发展、共同富裕、质量兴农之路，健全人居环境长效管护机制。通过5年的努力，我市的城乡差距进一步缩小，让农区、牧区、林区、垦区产业兴旺、生态宜居、乡风文明、治理有效、生活富裕。

——发展动力更加强劲。坚持需求导向和问题导向，全面深化改革，扩大开放，推动创新。加快新旧动能转换，深入实施创新驱动战略，推动机制、管理、技术和品牌创新。实施重大科技项目，建立以企业为主体、市场为导向、产学研用深度融合的技术创新体系，大幅提升自主创新能力。加快科技成果产业化步伐，不断提高科技创新对发展质量的贡献率。推动各领域改革落地见效，生态文明制度、农牧业产业化、旅游先行区、口岸经济、混合所有制经济等重点领域和行政管理体制等环节改革取得新成果,市场化程度提高，各方面制度更加成熟。深度参与“一带一路”建设，充分利用外接俄蒙、内联东北的区位条件，依托“两个市场、两种资源”，坚持联动周边、双向开放、多边合作，构建全域开放新格局，努力将呼伦贝尔建设成为欧亚大陆桥的重要枢纽，在建设国家向北开放重要桥头堡上取得新突破。中俄蒙合作先导区建设在先行、引导、示范方面取得新成果，满洲里国家重点开发开放试验区建设取得新进展。完善体制机制，加强区域合作，在农畜产品精深加工、旅游开发等方面深度参与东北振兴战略，加大对环渤海经济圈、长三角、珠三角等经济发达地区的招商引资、引智力度。在推动对内对外开放，引进资金、技术、人才等方面取得新成效。通过5年的努力，我市的改革不断深入，开放不断拓展，发展潜力充分释放。

——人民生活更加美好。牢固树立以人民为中心的发展思想，把增进民生福祉作为发展的根本目的，多谋民生之利、多解民生之忧，抓住人民最关心最直接最现实的利益问题，既尽力而为，又量力而行，一件事情接着一件事情办，一年接着一年干，不断满足人民日益增长的美好生活需要。公共财政支出向民生和社会事业倾斜力度进一步加大，居民收入与经济增长同步，中等收入群体不断扩大，高度关注弱势群体，人民群众生活水平不断提高。坚决打赢脱贫攻坚战，全面完成脱贫攻坚任务,现行标准下贫困人口全部脱贫，贫困旗市全部摘帽。落实就业优先战略和积极就业政策，鼓励创业带动就业，实现更高质量和更充分就业。坚持兜底线、织密网、建机制，全面建成覆盖全民、城乡统筹、权责清晰、保障适度、可持续的多层次社会保障体系。加快教育、医疗卫生、文化、体育、养老等社会事业发展，不断提高基本公共服务均等化水平。高水平承办第十四届全国冬季运动会。加强和创新社会治理，筑牢祖国北疆安全稳定屏障。建设“法治、平安、和谐”呼伦贝尔，依法打击和惩治违法犯罪活动，促进社会公平正义，人民群众合法权益得到切实保障。着力提升公民思想道德和科学文化素质，完善社会信用体系，创建全国文明城市、国家卫生城市、全国民族团结进步示范市，巩固民族团结进步、边疆和谐安宁的大好局面。通过5年的努力，人民群众的获得感、幸福感、安全感更加充实、更有保障、更可持续。

三、2018年主要工作

2018年是贯彻党的十九大精神的开局之年，是改革开放40周年，是决胜全面建成小康社会、实施“十三五”规划承上启下的关键一年。做好今年政府工作意义重大。

经济社会发展的主要预期目标：按可比口径计算，地区生产总值增长6%左右；一般公共预算收入增长6%；限额以上固定资产投资增长8%；社会消费品零售总额增长8%；城乡居民人均可支配收入分别增长7.5%。

生态环境的主要预期目标：草原综合植被覆盖度稳定在70%；新增活立木蓄积量0.2亿立方米以上；主要河流湖泊功能区水质达标率达到67%；污水集中处理率达到95%；可治理沙地土地治理率、受污染耕地安全利用率完成自治区下达任务；城市空气质量优良天数比例达到97%。

为实现上述目标，重点抓好以下十个方面工作。

（一）坚决打好三大攻坚战。要强化责任意识、风险意识，敢于担当，履职尽责。

打好防范化解重大风险攻坚战。加强对金融、债务、稳定等各类风险的评估，建立健全风险预警机制，制定系统的防范和化解风险方案。重点抓好财政、金融风险防范化解工作。强化政府债务管理，规范举债行为，落实政府性债务化解方案，如期完成化债任务。依法加大对非法集资、高利贷和不规范的互联网投资等违法行为的打击力度，严控金融风险。严格控制一般性支出，“三公”经费只减不增。

打好精准脱贫攻坚战。坚持精准扶贫、精准脱贫，确保1.4万贫困人口脱贫、4个区贫旗市摘帽。加大“五个一批”工程的实施力度，抓好产业扶贫、健康扶贫，保障住房安全，鼓励引导商业性、政策性、开发性、合作性等各类资金支持扶贫开发。注重扶贫同扶志、扶智相结合，发挥群众主体作用，增强贫困人口自我发展能力。健全精准扶贫工作机制，充分发挥驻村干部作用，抓好精准识别、精准退出，确保脱贫成果经得起检验。

打好污染防治攻坚战。持续推进中央环保督察反馈问题整改工作，确保年度整改任务如期完成。深入实施大气、水、土壤污染防治专项行动，全面落实“气十条”“水十条”“土十条”。启动新一轮土壤污染状况详查。实施第二次全国污染源普查，抓好工业、农牧业面源污染和矿山地质环境综合治理。建立重点排污企业基础数据库，强化动态监管。完成14座城镇污水处理厂提标改造工程。全面强化集中式饮用水水源地保护区域环境管理。新建16个地表水国家监控断面水质自动监测站。继续实施城市建成区10蒸吨以上非火电行业燃煤锅炉污染治理设施提标改造，10蒸吨及以下燃煤锅炉全部淘汰或改用清洁能源。

（二）大力推进生态文明建设。“绿水青山”就是“金山银山”，只有恢复“绿水青山”才能使“绿水青山”变成“金山银山”。

加强生态修复治理。对山水林田湖草进行系统综合治理。严格落实新一轮草原生态保护补助奖励政策，全年禁牧1687万亩，草畜平衡8671万亩。推进生态草牧业试验试点区建设。退牧还草140万亩。退耕还草10万亩。推进天然林保护、森林生态效益补偿、三北防护林、森林质量提升等重点生态工程，巩固扩大天然林商业性停伐和退耕还林成果。完成绿化造林75万亩。继续实施沙区综合治理，建立草原沙化监测系统。实施呼伦湖综合治理二期工程，做好环湖整体生态系统综合整治，启动呼伦湖周边300万亩原生态草原和游牧文化保护区建设。坚持依法管理、综合治理与科学监测并重，水域面积稳定在2050平方公里左右，水质稳步向好。加强河流流域综合治理，深入落实河长制、湖长制。抓好国家湿地公园建设。建立绿色矿业发展示范区，推动绿色矿山建设。整体提高各级各类自然保护区建设管理水平。

推动生态文明制度建设。依法严厉打击各类破坏资源环境行为。严格落实国家主体功能区规划。继续做好自然资源资产负债表编制和领导干部自然资源资产离任审计试点工作。争取国家将呼伦贝尔列为森林草原碳汇交易和排污权排放、排污权交易试点地区。全面落实生态文明建设目标评价考核办法，建立资源有偿使用、生态文明评价考核、生态环境损害责任终身追究等制度。完善生态环境监管制度，强化环境质量网格化监管。加强生态文明宣传教育，提高全民生态环保意识，倡导绿色低碳生活方式。

（三）促进工业转型升级。传统产业不等于落后产业，要抓好转型升级；新兴产业要积极培育、大力支持，加快形成新的增长极。

加大传统工业改造提升力度。煤炭行业加快神宝公司、大雁公司扩能技改；化工行业支持东能、金新提质增效；有色金属行业推动驰宏矿业锌合金生产线投运、荣达矿业深部资源接替技改和中金公司含铜废石综合利用项目；电力行业支持伊敏煤电电厂超低排放改造项目，争取牙克石汇流河热电联产项目机组改型方案获得国家批复，积极推进呼伦贝尔至河北南网电力外送通道建设；木材加工行业加快满洲里装配式木结构建筑生产加工基地建设；装备制造业实施华德、蒙拓农牧业机械智能化升级改造。

培育壮大新兴工业。启动实施光伏扶贫项目，争取将光伏发电领跑者示范基地一期宝日希勒沉陷区50万千瓦光伏项目列入国家、自治区规划。加快沃丰10万吨液体肥、30万吨生态肥项目建设。实施日冕公司、荣嘉公司3万千瓦生物质发电项目。争取光伏发电“领跑者”示范基地规划纳入国家实施方案。支持阜丰集团拓展玉米生物科技产业链。推进大庆博润30万吨乙醇等生物科技项目。加快海拉尔大数据中心一期项目建设。

提高园区发展能力。充分发挥园区发展平台作用，释放工业集聚发展效应。加快呼伦贝尔经济技术开发区、岭东工业园区、伊敏工业园区等重点园区建设。加大精准招商力度，优化服务环境，完善配套政策，大力发展绿色、节能、环保产业。

（四）做精农牧林业。深入推进农牧业供给侧结构性改革，以“名优特精”为发展方向，变数量增长为质量效益增长。

调整优化种养结构。农区玉米种植面积稳定在750万亩，大豆种植面积扩大到900万亩，新增设施农业3万亩。扩大马铃薯、甜菜种植面积。加快发展生态节水型农牧业，新增高效节水灌溉农田60万亩。实施黑土地保护10万亩。耕地轮作65万亩以上。引导牧区走草畜平衡、少养精养之路。提高农区养殖业水平，扩大农区肉牛、生猪养殖规模。林区重点抓好蘑菇、木耳、苗木、中草药、蓝莓、狐、貂等特色种养业。

提高产业化水平。培育壮大呼伦贝尔农垦集团、阜丰集团、伊赫塔拉等龙头企业。支持晟通糖业100万吨甜菜制糖、禾牧阳光肉牛良种繁育、呼伦贝尔肉业集团肉牛繁育等产业化项目。做强玉米生物科技、健康食品、蒙中医药产业基地。大力发展马铃薯种薯繁育基地，支持组建薯业集团。加快草牧业科技示范园区建设。结合推进退耕还林还草，苜蓿种植面积达到30万亩以上。全年农畜产品加工转化率达到63%，农企利益联结机制达到58%。健全农牧业社会化服务体系。引导农牧业清洁生产，促进形成绿色种植、循环种养新格局。

加大品牌建设力度。围绕肉牛、肉羊、蘑菇、木耳、蓝莓、药材等一批绿色优质农畜林产品，打造更多的“呼伦贝尔”名优特精区域公用品牌。加强农产品质量安全监管，“三品一标”认证增加20个。完善农畜林产品市场营销体系，大力推进“互联网+”，实现种得好、养得好向质量好、效益好转变。

（五）加快现代服务业发展。以原生态、国际化、全域游的旅游业为引擎，带动服务业发展。

做优旅游业。以“大草原、大雪原、大花园、大族源”为主题，推动旅游与自然生态、民族民俗、文化体育的深度融合。加快旅游景区景点的提档升级，打造一批自然景观和地域文化深度融合、具有较大影响力的精品景区，重点抓好呼伦贝尔大草原旅游区、额尔古纳湿地5A级景区创建工作,开发草原森林旅游观光列车系列项目，完善蒙古之源·蒙兀室韦民族文化园、拓跋鲜卑历史文化园、白音哈达草原度假区、两河圣山等景区功能，实施套娃广场二期项目建设。开展“田园综合体”建设，规划建设一批“小特精”的农区特色村落、牧区庄园牧场、林区森林康养、垦区特色风光等民俗体验式旅游项目。组织开展中国·鄂伦春国际森林山地运动节、中国冰雪那达慕等冬季旅游系列活动。围绕“商、养、学、闲、情、奇”和“吃、住、行、游、购、娱”，创新特色旅游产品，满足游客多元化需求。完善和优化呼伦贝尔草原经典游、呼伦贝尔多民族风情体验游、呼伦贝尔岭东南风光民俗游等精品旅游线路。加快旅游标识、旅游驿站、旅游厕所、救援救助中心等服务设施标准化建设,重点抓好国家旅游局数据中心呼伦贝尔边境旅游数据基地项目。加强旅游执法，建立健全旅游企业诚信机制，清理整治重点区域、公路沿线两侧“小散乱污”景点景区。

做活现代服务业。坚持传统与现代商贸物流并举，加快海拉尔生活性物流园和阿荣旗、扎兰屯商贸物流园建设。推动物流信息化，建设商贸物流综合信息服务平台。确保满洲里口岸集团国际物流中心和中俄农产品贸储项目落地。提高餐饮、住宿、娱乐、购物等传统服务业水平。大力发展健康养老、金融会展、电子商务、社区家政服务、信息咨询等新兴服务业。支持分享经济、跨境电商等新业态、新模式发展。

（六）统筹推进城乡建设。抓重点、强弱项、补短板，提高基础设施保障经济社会发展的支撑能力。

优先发展中心城区。加强市区共建，注重规划引领，完成呼伦贝尔市城市总体规划和系列专项规划的修编工作。围绕国际化“草原音乐名城”“冰雪运动名城”“生态旅游名城”建设和“做活老城区、做优新城区、做亮文化区、做特民族区、做大外环生态圈”，高标准制定完善“三城”规划。抓好历史文化街区、国际会展中心、草原文化国际交流中心等项目建设。启动黑羊站水厂12万吨处理能力扩能改造，完成红花尔基水库引水工程建设。继续实施老旧小区综合改造。加强城市环境综合整治，提高城市精细化管理水平，继续推进“三清”治理，营造洁净、有序、文明的城市环境。

完善城镇功能。突出农、牧、林、垦、口岸等不同类型区特点，统筹推进各旗市所在地、重点镇、中心镇建设和管理。抓好莫尔道嘎镇产城融合特色小镇和扎兰屯市、大杨树镇国家新型城镇化试点建设。实施背街巷道、丁字路、断头路改造56条，建设改造停车场45座，完成供水、供热、污水等老旧管网综合改造144公里，新建改造水冲式厕所150座。启动根河、额尔古纳等地新热源建设。强化城镇管理，改善市容市貌和环境卫生，提升绿化、亮化、美化档次，规范道路交通秩序，依法严厉打击违建，提高小区物业管理综合服务水平。

建设美丽乡村。实施乡村振兴战略，高水平规划建设一批示范村屯嘎查。选好致富带头人，充分发挥大学生村官、驻村干部、退伍军人的带动作用，助推“一村一品、一乡一业”，把“小特精”产品做成大经济。积极引进社会资本进入农牧林垦区，推动农田、草牧场向先进生产要素集中，适度扩大规模经营，提高专业化合作水平。壮大集体经济，增加农牧民收入。通过种养结合、生态循环，积极构建田园、草原、森林、河湖生态系统。实施农牧林垦区人居环境综合整治，建立城乡统筹垃圾处理长效机制。推进人畜分离，落实好“厕所革命”具体任务。发展基层协商民主，强化村务公开，完善一事一议制度。加强乡风文明建设，丰富农牧民文化生活。

加强基础设施建设。市政基础设施投资50亿元，推进市政基础设施建设及改造。铁路投资5亿元，推动阿莫铁路建设，开工岭东工业园区铁路专用线，推进齐海满城际列车等项目前期工作。公路投资38亿元，加快海拉尔—满洲里、海拉尔—伊敏等11条重点公路及农村公路建设，积极推进博克图—紫沟、海拉尔—阿木古郎等5条重点公路项目前期。民航投资2.6亿元，加快陈旗、满归通用机场和海拉尔东山机场平滑道项目建设。水利投资12亿元，完成嫩江干流治理主体工程，推动Z866项目建设, 开工阿伦河、甘河、根河、激流河治理项目。电网投资12.2亿元，加快海拉尔—牙克石—扎兰屯岭东500千伏输变电工程、海北—岭东—冯屯500千伏输变电工程建设。继续实施“宽带呼伦贝尔”工程。

（七）增强改革开放创新能力。坚持问题导向促改革，融入国家战略促开放，实施创新驱动促发展。

推动改革落地见效。继续深化“放管服”改革，巩固提升商事制度改革成果，实现企业注册登记全程电子化，以政府职能转变激发市场活力。深化农村牧区集体产权制度改革，全面推开土地草牧场确权工作，落实农村牧区承包地“三权”分置制度。深化不动产统一登记制度改革，完善不动产登记制度体系。加快地方国有林场改革，做好“三供一业”等社会职能剥离后续工作。支持大兴安岭重点国有林管理局探索建立国有林区管理新体制和经济发展新模式。推动呼伦贝尔农垦集团建立现代企业制度，完成呼伦贝尔农垦集团剥离办社会职能改革。加快财税金融体制改革，推进实施绩效管理，建立现代财政制度。深化投融资体制改革，完善金融服务体系，发展多层次资本市场，增加直接融资比重。推动普惠金融发展，增强金融支持实体经济发展的能力。发展混合所有制经济，鼓励民间资本参与国企改革，推动投资主体、投资渠道多元化。树立抓营商环境就是抓生产力的理念，支持非公经济健康发展，研究制定和积极落实各项支持政策和措施，为民营企业投资兴业搭建各类服务平台，重视本土企业家队伍建设。

提高开放水平。积极融入国家“一带一路”战略，加快中蒙俄经济走廊建设。用足用好自治区《关于支持呼伦贝尔中俄蒙合作先导区建设的若干意见》。加快满洲里国家重点开发开放试验区建设，打造中俄商品交易中心。推动“海赤乔”国际次区域合作取得实质性进展。推进中俄中蒙跨境经济合作区、跨境旅游合作区和额尔古纳边境旅游试验区申建工作。力争额布都格互市贸易区通过验收，推动阿日哈沙特互市贸易区获批。完成额布都格、阿日哈沙特口岸常年开放验收。推动室韦—奥洛契口岸援建工作，室韦口岸实现客货双通。做好额布都格—巴彦呼舒口岸2号界河桥前期工作。加快口岸电子信息化建设。推动“海满阿”区域协同发展。加强与东北经济区在基础设施、旅游、生态、能源供应等方面的深度合作。加强对环渤海、长三角、珠三角经济发达地区的招商引资、引智力度。

增强创新能力。深入实施质量强市、品牌兴市战略。发挥企业的创新主体作用。加强产学研用深度结合，深化与国内高校和科研院所的务实合作。充分发挥院士工作站、工程技术中心、新型研发机构等平台在科技创新成果转化的引领作用。完善科技创新机制。坚持创新创业结合、孵化转化结合，推动商业模式和业态创新。加大科技投入，吸引国内外高层次人才和团队在农牧业、草牧业、生态环保、生物科技、蒙中医药等领域开展科技攻关和技术推广。

（八）切实保障和改善民生。坚持以人民为中心的发展理念，让人民群众共享发展成果。

实施重点民生工程。推进创业就业工程，多渠道开发就业岗位，建设一批示范性创业园和孵化基地。全年城镇新增就业3万人，农牧民转移就业6万人，做好高校毕业生、转业军人等重点人群就业工作。抓好保障性安居工程。投资60亿元，城市棚户区改造开工18360户。提高社会保障能力，实施全民参保计划，扩大参保覆盖面，落实机关事业单位养老保险和城乡低保标准及特困供养标准的合理增长机制。完善统一的城乡居民基本医疗保险、大病保险和失业、工伤保险制度。

加快社会事业发展。提高各级各类教育发展水平。办好义务教育、学前教育、特殊教育、民族教育。推进呼伦贝尔学院转型发展，支持呼伦贝尔职业技术学院、扎兰屯职业学院加快发展。促进文化繁荣进步。加大公共文化服务设施建设力度。活跃文艺创作，加强对外文化交流。强化文化阵地建设和管理，落实意识形态工作责任制。大力培育和践行社会主义核心价值观。继续开展全国文明城市创建工作，着力提升市民文明素质。实施健康呼伦贝尔工程，持续深化医改，健全公共医疗服务体系，提升基层卫生服务能力，加强全科医生队伍建设和中蒙医人才培养。抓好蒙中医药健康产业园项目建设。推动牧区小药箱全覆盖。大力开展爱国卫生运动，启动国家卫生城市创建工作。落实好计划生育各项政策。推进全民健身和竞技体育同步发展，实施旗市区所在地“10分钟健身圈”及乡镇苏木、嘎查村体育设施全覆盖工程。做好第十四届全国冬季运动会筹备工作，加快海拉尔、牙克石、扎兰屯比赛场馆和配套设施建设。加快各旗市区科技馆建设。落实好粮食安全市长、旗市区长责任制。关注老龄事业，推动医疗与养老服务融合发展。支持红十字事业和公益慈善事业健康发展。统筹推进市场监管、气象、人防、地震、档案史志等工作。

（九）加强和创新社会治理。构建和谐社会，筑牢北疆安全稳定屏障。

全面推进依法治市，扎实开展“七五”普法，加强公共法律服务体系建设，切实增强全社会法治观念。探索创新社会综合治理模式，建立健全公共安全体系。深入推进平安呼伦贝尔建设，健全完善立体化社会治安防控体系，严厉打击和惩治黄赌毒黑拐骗等违法犯罪活动。抓实信访维稳工作，健全依法维权和化解纠纷机制，妥善解决群众合理诉求。严格落实安全生产责任制，推进风险管控、隐患排查双重预防体系和信息化建设，坚决遏制重特大事故发生。提升食品药品监管能力，保障群众饮食用药安全。加强森林、草原防火工作。完善防灾救灾减灾体系建设。全面落实党的民族政策，提高民族宗教事务治理法治化水平，加强少数民族语言文字的普及和推广使用。争创全国民族团结进步示范市。支持莫力达瓦达斡尔族自治旗、鄂温克自治旗组织好建旗60周年庆祝活动。推进国防动员、人防和军警民边防体系建设，提升边境综合防卫管控能力，维护边疆安宁稳定局面。

（十）打造人民满意政府。人民政府让人民满意是我们的最高追求。

提高政治站位。不断增强“四个意识”，坚定“四个自信”。旗帜鲜明讲政治，深入学习贯彻习近平新时代中国特色社会主义思想，全面落实中央、自治区党委、政府和市委的各项决策部署，把党的十九大精神转化为政府工作的强大动力和生动实践。

坚持依法行政。严格执行市人大及其常委会的决议决定，自觉接受人大及其常委会的法律监督、工作监督和人民政协的民主监督。政府工作人员要按照法定权限和程序行使权力、履行职责，确保法无授权不可为，法定职责必须为。严格规范行政执法行为，做到行政合法、程序正当、权责统一。以群众利益作为决策的基本出发点，认真汲取各方面对政府工作的意见和建议。加强调研论证，强化风险评估，坚持合法性审查，完善公众参与、专家咨询和政府决策相结合的工作机制。坚持重大行政决策集体研究，严格执行“三重一大”事项决策程序和规则。

坚持高效施政。民之所望，施政所向。政府公务人员要说实话、谋实事、出实招、求实效。严格落实领导干部联系和服务群众制度，推动机关干部深入基层、服务群众。创新公共服务举措，大力实施“互联网+政务”,优化网上办事大厅功能，扎实推进政务服务体系向基层延伸，提高办事效率，实现“信息多跑路，群众少跑腿”。提高行政服务效能，进一步精简行政审批流程，有效降低制度性成本。构建“亲”“清”新型政商关系，营造良好发展环境。

坚持从严治政。严格落实管党治党主体责任和“一岗双责”，把从严治党责任落实到从严治政中。严格督政问责，重拳整治慵政、懒政、怠政。加强对权力运行的制约和监督，让权力在阳光下运行。强化对资源开发利用、项目招投标和政府采购等重点领域的廉政风险防控，加强行政监察和审计监督。严格落实党中央八项规定实施细则、自治区和市委的配套规定，持续纠正“四风”。严厉惩治违规违纪行为，坚决纠正侵害群众利益的不正之风。

各位代表，过去五年创造的发展业绩需要倍加珍惜，未来五年描绘的宏伟蓝图更加催人奋进。让我们紧密团结在以习近平同志为核心的党中央周围，在自治区党委、政府和市委的坚强领导下，守望相助，团结奋斗，扎实工作，一往无前，奋力谱写决胜全面建成小康社会的呼伦贝尔新篇章，为“建设亮丽内蒙古，共圆伟大中国梦”而努力奋斗！
